# New York Odontological Society

**Published:** 1892-07

**Authors:** 

**Affiliations:** New York Odontological Society


### Reports of Society Meetings.

### NEW YORK ODONTOLOGICAL SOCIETY.

A REGULAR meeting of the New York Odontological Society was held on Tuesday evening April 19, 1892, at the New York Academy of Medicine, No. 17 West Forty-third Street, with the President, Dr. Woodward in the chair.

The minutes of the previous meeting were read and approved.

Dr. J. Allen Osmun, of Newark, N. J., then read the following paper, entitled "Some Observations on the Use of Copper Amalgam."

(For Dr. Osmun's paper, see page 515.)

### DISCUSSION.

*Dr. Hart.*—I was rather surprised to hear the essayist state that the working qualities of Weygant's amalgam and Dr. Russell's preparation were the same. While I have worked with both, I had some little success with Weygant's, and none at all with the other. I attributed my failures to the fact that Russell's preparation was turned on the market with such a small quantity of mercury. In nearly every instance, before I could get the amalgam to work, I heated the preparation; Weygant's would bubble freely before it was brought to such a high degree of heat. I must also take exception to the statement made that the copper amalgam was equally good in approximal fillings as in buccal cavities. The buccal fillings seem to last, and in very few instances did they fail to crystallize. I have had some failures, like those mentioned by the essayist, where the fillings could be cut out freely with a spoon excavator, while in the same mouth other amalgams were doing good work. It leaves one in rather an uncomfortable position, because the operator does not always know where to select the case in which to use it. Some fillings make you think that you could introduce it in all. The patients are seen at some future time, and the fillings are found a dismal failure.

*Dr. Bogue.*—No doubt there are many gentlemen present who have used it more than I have, although I doubt whether any have longer. The last speaker's remarks would seem to indicate that he, too, had come across various methods, and possibly various preparations. I do not think we sufficiently well define ourselves, when we are speaking of these materials. For example,—we all know what a galvano-plastic plate is, and have looked at copper plates thrown down by the action of a galvanic battery with some amusement; but our Newark friends have discovered that if a solution of gold is sufficiently attenuated, very weak, and the battery is sufficiently weak, gold plate can be cast down so as to produce an almost uniform thickness of great density and strength. That same truth is applicable to copper amalgam. When the solution from which the amalgam is made is exceedingly attenuated, and is thrown down gradually, the product is a fine amalgam which will take the imprint of the cavity or the end of the fingers very accurately. If it is allowed to harden, and is then softened again by heat, and squeezed, and again hardened and again softened and thoroughly squeezed, you will get a product which will go into a cavity very accurately, and will produce an amalgam upon which no excavator that I have come across will have any effect. It does not readily waste. Even the burr will not take it out sometimes. Copper amalgam made by some of the rapid galvanic processes does not seem to be fine-grained enough. It is gritty, and you cannot get a good filling. I do not know why. The only copper amalgam of which I know anything at all is the one made after Rogers's formula, and is made after the process of which I speak. The galvanic current is exceedingly weak. It took several days to deposit copper enough to answer my purposes in the office. That copper amalgam, when the mercury had been pressed out several times, has done excellent service.

I do not use it often, but it is good for the restoration of breaks in the back of the mouth. It has answered a purpose that nothing else than palladium has accomplished. I would like to tell of something that has caused me much trouble and cost some money to learn. There is to-day but one house in the world where you can buy precipitated palladium. That is Johnson, Matthey & Co., 79 Hatton Garden, London. I asked Ash for some, and inquired for the address of the firm, but he would not give it to me; but said he would procure it and be responsible for it. He did procure some, but I could not use it; it was very brittle, and it was with difficulty that I could amalgamate it with mercury. When I returned it, I had a conversation with him, the moral of which is that there are various kinds of palladium. I do not know whether you are ever required to use it, but if so, you must be careful to get the right kind.

A gentleman has just asked me if Sullivan's and Rogers's copper preparations are the same. Sullivan's was made in Germany many years ago. Rogers's was made in London within the last fifteen years. Sullivan's is exceedingly dirty and does not answer the purpose for me.

*A Member.*—You speak of softening the amalgam. What do you mean by that?

*Dr. Bogue.*—Heating it until it sweats mercury, and then working it in the hand or in the mortar, and then letting it harden, again heating it until the mercury sweats, then rubbing it in the mortar, and then squeezing all the mercury through a chamois-skin and again letting it harden. Do that three or four times, and then let it harden. The result is a product that is different from any we have.

*Dr. Hart.*—Did this copper amalgam that you speak of waste very much?

*Dr. Bogue.*—I do not know that it wastes very much. I have seen some twenty-one years old. It does waste slightly, but so little that it is hardly perceptible.

*Dr. Watkins.*—I dislike to disagree with my friend, Dr. Osmun, but I have had experience with copper amalgam to my sorrow. I used it a great deal at one time, and regard Russell's as finer and cleaner than any of the others. The amalgam will apparently appear to work well, and will seem to give perfect satisfaction. In a brief period the filling is bright but wasted, and in such cases it has been my experience that the tooth is exceedingly sensitive around and under that filling. I have had a different experience from some of the gentlemen, inasmuch as instead of finding the fillings soft, so they could be taken out with a spoon excavator, they seemed to me to be as hard as steel. It is difficult to make the two things agree. I have come to one conclusion in regard to this amalgam, and that is, never to use it except in cases where we have very soft, white teeth, where the saliva is inclined to be acid. Take, for instance, teeth which are pearly white, and in which the decay is rapid. I have seen many where it has been in such mouths for several years, and doing beautifully, as black as ink, the lines perfect, no sign of decay. I met a case of that kind a few days ago, and I was pleased to see how beautifully the teeth were being saved; but I think such are exceedingly rare.

The subject to my mind is still in doubt. There are so many cases where fillings have been put in at the same time and apparently under the same conditions, where one will be good and the other will have left the cervical wall, so that an excavator can penetrate the material. Take two approximal cavities, where one filling will be good, black, and not deteriorated. Perhaps in the adjoining filling one part will be good and the other part wasting, and yet bright in color. It seems to me an experiment, and the safest thing is to let it entirely alone, with the exception of the few cases under the conditions which I mentioned.

*Dr. Howe.*—From a limited use of copper amalgam, I came to the conclusion that it was a very uncertain material, and I have not employed it for fully two years. I have had some success in making good permanent fillings, but very many more failures. Whatever antiseptic influence it has, it does not prevent decay from beginning and progressing directly in contact with it. I failed to find in it any constant qualities that were desirable, and when I became convinced that I could not make fillings that I could rely on, I abandoned its use.

*Dr. Bogue.*—Before Dr. Howe's remark has passed from memory, I want to speak of a little incident that has occurred to me. Most of us use pepper when we lay our clothing away in the spring-time, so the moths will not get at it. Not long since, I opened a box of Darby's capsicum pads, and found they were all moth-eaten, apropos of copper amalgam.

*Dr. Francis.*—I came here not to impart any information in regard to copper amalgam, but rather to learn something about it. When I first heard that such a filling was in use I was much prejudiced against it, believing it an improper material to put in the mouth, and it was a long time before I could persuade myself to try it. In the mean time I had heard and read much concerning it, also had repeated opportunities of seeing and examining fillings of this material in a number of mouths where it had been employed.

I noticed cavities filled with it where the walls were very frail and yet the teeth were not discolored in the least; the discoloration being confined to the exposed surfaces of the amalgam. I have, however, seen some teeth badly discolored by the filling; and other cases where the fillings assume a frosty white appearance and the teeth not discolored, yet looking somewhat worn. I must admit that copper amalgam has been a puzzle to me even to the present time.

I have heard much said of its preserving properties, and feel convinced that there are many cases where it has preserved cavities fully as well, if not better than anything else. I mean such cavities as are very difficult to fill with gold. I have not witnessed many failures, but have seen enough of them to make me hesitate before using it; and hardly know yet whether to recommend it or not. I think, however, that in particular cases, for instance on the buccal or even the approximal surfaces of the back teeth, it may be used advantageously. I have, in several instances, had occasion to remove copper amalgam fillings, but they were so hard as to render the operation difficult. I have seen cases where the fillings work perfectly smooth and the cavity margins well preserved. I have also noticed successes and failures in the same mouths. Whether or not these varied conditions are due to the manipulation or preparation of the amalgam, I do not know. In cases where fillings appear worn away, I have thought the filling had been overheated. Like others, I have used it with success, but with some failures. The same rule will apply to other stoppings.

*Dr. Rich.*—I have had no experience with copper amalgam, as I have never used it, and cannot, therefore, speak about it from the stand-point of personal experience. I have seen quite a number of fillings constructed of it that were not preserving the teeth, but as I did not know anything about the preparation of the cavity, or the amalgam, or how it was introduced into the cavity, I had no means of forming an intelligent judgment about it. The general impression in regard to it made upon my mind by those fillings I have examined would be against its use.

I am surprised to hear so many dentists say they have used it, and yet acknowledge that they knew almost nothing about its composition and preparation. Dr. Bogue's experience is that in his hands it is generally successful, that as he prepares and uses copper amalgam it will preserve the teeth, and he seems to know more about its composition and preparation than any other person who has spoken of it to-night. Others, who have given us their experience with it, say that in their hands it is almost always a failure, but those who speak of it in this way do not seem to know much about its preparation or properties.

*Dr. McQuillan.*—At one time I used considerable copper amalgam, but for the last eighteen months I have not inserted one filling of it. While my successes have been greater than my failures, the failures have been very lamentable. I have had a success on one side of the mouth, and a failure on the other. I, therefore, came to the conclusion that it is too unreliable for me to use. I can get a better result on buccal surfaces with gutta-percha. I think my patients have swallowed enough copper amalgam to satisfy me, and I shall not use any more.

*Dr. Perry.*—I do not know much about copper amalgam. I do know it is very unreliable, and do not use it; and wish I never had, and the greatest mistake of my professional life was made when I did. I think, however, I should be partly excused for having taken it up. I am going to put in a plea for myself, as well as for the profession, because, if you think back for ten years, you will remember that there has been considerable said about it, coming partly from the other side, and said in such a way as to leave the impression upon the minds of the profession at large that it was a material which did not shrink. Of course, for many years we have been searching for just such a material. When some of us saw some fillings in different mouths that were looking well, and not having the appearance of shrinkage, I think we were all justified in concluding we had the material we wanted, and had something we could depend on. We had heard of its success, and had seen cases where it had succeeded; but not a word yet about the failures. In my own experience, the first series of cases I saw were very remarkable indeed. Dr. Darby sent a patient to me with a number of those fillings, and I think those were the first ones I had ever seen that I really knew were copper amalgam fillings. I was profoundly impressed with the appearance of these, and, never having any suspicion of danger of waste at the cervical margins, or mechanical wear on the surface, and having no hint of the unusual danger of discoloration in dead teeth, I was ready to commence its use. Dr. Rich, of course, is right in saying that we should not use anything of which we are ignorant; but we must do the best we can, and we felt that we did know something about this amalgam. The danger of discoloration we did not suspect, for I have no knowledge of any hint having been given in the literature on the subject; but this I found to my regret subsequently.

I have some black monuments standing in the mouths of patients, which I would give a great deal to efface. There is no hope of changing them, because it cannot be done. Copper amalgam does not affect the living tissue of living teeth, but it invariably discolors the bodies of dead teeth themselves. It seemed to be a great point to have something with which frail shells could be restored to usefulness. I felt that at last we had a material that would be of great value in the matter of restoring the shapes of the teeth, and thereby keeping the occlusion perfect. I suppose I am rather too enthusiastic; when I see what appears to be a good thing, I adopt it. In the early years of my practice I made a mistake in filing and separating the natural teeth. But I learned something by that experience, and I believe my patients have since profited by it. I must not be too sweeping and positive in the condemnation of copper amalgam, because it has a certain quality which some day may be made available. I must say I have seen a few cases where it has done well. Certain teeth with small fissures on the grinding surfaces can be benefited by it, and I think, in the teeth of children, it can be made available, because we do not expect long use in temporary teeth. It is soft and manageable, when nicely prepared, and easily applied. Some of our friends who have never used any amalgam at all have had much to say in reference to the effect of mercury. I do not know if I am quite right, but I think that applies more particularly to men who have homœopathic leanings. What will our homœopathic friends say to the question, What becomes of the mercury?

*Dr. Ives.*—I only inserted one copper amalgam filling in my life. I regret the broken instruments destroyed in removing the *débris*. I do not think that any class of teeth can be as well saved by good ordinary amalgam as by copper amalgam.

*Dr. S. E. Davenport.*—It is a satisfaction to me to know that gentlemen possessing the attainments of our essayist are devoting their attention to the study of this question, for I feel that there are qualities inherent in copper amalgam which are very useful, and if a way could be found to make them always available, we would have a most valuable material. My experience does not differ very much from that of the other gentlemen who have taken part in the discussion, and I do not think anything would be gained by referring to it in detail. I have recently had several opportunities of observing copper amalgam fillings in the mouths of patients of some of the best-known English dentists, and I think their proportion of successes is not greater than ours, for I found but few of the fillings doing good service. I began to use copper amalgam about eight years ago, and for several years I used it for certain cavities in soft teeth with considerable satisfaction; but for the past two or three years I have used it but seldom. The amalgam which has been referred to by Dr. Bogue, prepared after the Rogers method, seemed at first to be almost universally reliable, but recently the quality has evidently deteriorated. I have had the pleasure of seeing in the mouths of some of Dr. Bogue's patients copper amalgam fillings, made probably fifteen years ago, which were almost perfect, and certainly doing as well as any filling possibly could have done under the circumstances, for, of course, they were not used in a high class of tooth-substance. I think, however, if Dr. Bogue should go a little more fully into his personal experience in the matter, he would tell us that he does not use copper amalgam frequently now, and has less confidence in it than he had some years ago.

*Dr. Bödecker.*—I am sorry I was not here in time to hear the paper and the discussion by some of the members, but I briefly state some of the experience I have had with copper amalgam in the past few years, although I have seen a great deal of it during the time I was in England. The gentleman whom I was with had a great deal of faith in copper amalgam, and claimed to obtain better results with it than with any other he used. This amalgam we made ourselves. In this country I have not employed any in practice until four or five years ago. I commenced to use it when it became famous here, so to speak, and I very soon found that there were many failures on account of shrinkage and wasting. About three years ago Dr. Herbst, of Bremen, wrote me a letter about using amalgam, saying that he had very wonderful results if he added to it, before putting it into the tooth, a little fine silver-foil. I tried that, and it worked as described,—namely, that the edges will stand beautifully, and there will almost be no wasting. At that time I made some experiments in glass tubes, and filling them with carmine, but I never saw any of the fluid between the tube and the amalgam. I tried the same experiment with copper amalgam, and I was surprised at the success. The discoloration of the copper amalgam which we usually observe is almost entirely gone, and it gets very much harder and wears better. It is done in the following way: When the amalgam is heated very carefully and put into the mortar, a little mercury is added, and the amalgam crushed and rubbed thoroughly; then to about eight grains of copper amalgam I add one leaf of the ordinary fine silver-foil which you obtain from any gold-beater. Then I put it into the tooth with the Herbst burnishers, and I must say I have not seen a failure around the edges, or much discoloration. Without the addition of silver to the copper amalgam I have seen more or less discoloration and wasting of the edges.

*Dr. Rich.*—May I ask Dr. Bödecker the weight of the silver-foil he used?

*Dr. Bödecker.*—I have no scales sufficiently delicate to weigh it. It is the fine silver leaf the gold-beaters make, which is used for silvering. It is not a quarter of a grain in weight. It dissolves instantly in the mercury.

*The President.*—We have received several communications from gentlemen who are not able to come to-night. The editor will oblige us by reading some of them.

*Dr. Dwinelle.*—Before the gentleman reads those papers, I will venture to volunteer a few remarks. I have had very much the same observations that others have expressed here to-night in regard to copper amalgam. I have been enabled to deduce certain conclusions, but I have not been an advocate of it, and have not used it to any great extent. I have employed other materials which have answered every purpose that I could wish for. I think one great cause of failure has been that these have not been properly prepared. I have a method of preparation by which I am assured of a very good degree of success, and that is, in washing my amalgam. Take the granulated amalgam,—that is, in grain form,—and if it has been on hand any length of time, it has become exceedingly oxidized, especially if it has been in the vicinity of sulphur. Old amalgam will waste to a considerable extent. I have had it waste to at least one-half its bulk. I wash it in carbonate of soda, and often wash away one-half of its substance in bulk; then I carefully prepare it and squeeze out the excess of mercury, and in that way have no trouble, especially in discoloration. I think that the old-fashioned amalgam is as good as any can be, copper amalgam not excepted. I can show fillings twenty-five or thirty-five, and perhaps forty-five years old.

*Dr. Francis.*—I never have felt sure of amalgam of any kind. I have employed it as others have. I am not even sure of gold fillings, because I have seen many crumble away.

*Dr. Dwinelle.*—In many cases of frail teeth, gold is perhaps the worst material to put in. I have filled frail wisdom-teeth many times to the best of my ability, and have failed every time, but when filled with amalgam, they succeeded, and they are there to-day.

On motion of Dr. Hart the written discussions were made part of the transactions.

W. D. Miller thinks it of great value, but its faults of wearing and washing out are serious.

J. N. Farrar believes that for preserving teeth it is equal, and in many cases superior, to any other amalgam. The great objection to its use is its color.

E. T. Darby: Does well on masticating surfaces. Not so well on proximate surfaces. The same material, worked in the same manner, will produce different results in the same mouth.

I. B. Davenport: When it turns and remains black, he concludes it to be the best material for saving deciduous and soft permanent teeth.

W. W. H. Thackston says he has not used it, but his observation of it is unfavorable.

W. H. Robbins formerly employed it freely, has since removed all,—some thousand fillings,—and does not use it now. Is sorry he did use it.

F. N. Seabury never used it.

J. L. Williams has considered it undesirable, and has been saved from much "experience" with it.

D. M. Clapp, from limited experience, finds numbers of failures out of all proportion to successes. As a rule, does not preserve from decay better than ordinary amalgam. Stains teeth badly.

C. A. Marvin has never used it.

C. S. Wardwell, from limited experience, finds it objectionable, and has ceased using it.

E. J. Niles considers it undesirable, on account of its wasting.

R. R. Andrews considers it unreliable. Washes out.

H. B. Noble used it sparingly for two or three years. Found nothing to commend it over other amalgams.

Sir Edwin Saunders used it some years ago, when in practice, "as a filling for buccal cavities for lower molar teeth, where it seemed to do well;" was apparently "free from shrinkage, but not hard enough for the attrition of mastication."

Geo. A. Maxfield has had favorable results from its use, but failures have occurred in a few mouths where the fillings hollowed out at centre, and in each case this has occurred in mouths where the teeth were free from salivary calculus; attributes many of the reported failures to the material being poorly made.

Louis Jack finds it has been of value in some cases, yet in many more, results have been so indifferent he felt compelled to abandon its use. Results have differed greatly from causes which do not appear, although the material was the same apparently, and prepared in same manner. Suspects possibility of serious consequences following the imbibition of the wasting material. Has not found the material prevents the recurrence of caries.

C. V. DuBouchet believes the merits of copper amalgam due to its apparent non-shrinkage, capability of hardening tooth-structure, and possibly its germ-destroying properties.

H. C. Quimby has observed many teeth badly stained by its use; thinks that it may have a value, but its unsightly appearance has deterred him from using it; thinks other amalgams equally preservative.

J. S. Latimer used it about a year; its unsightly appearance and slow setting and its rapid wasting caused him to cease using it.

W. A. Potter says that its color is so objectionable that his use of it has been very limited.

C. P. Stockwell considers it "preferable, all things considered, to any other material, . . . in a very narrow field."

C. S. Stockton has "practically ceased the use of copper amalgam because of its uncertain results."

C. N. Peirce: From a limited experience, its use "has not been satisfactory, because of its frequent disintegration at cervical borders."

J. A. Woodward: Its use has been limited to small approximal cavities of bicuspid and molar teeth. It has failed to arrest decay as frequently as other amalgams that I have used for similar cavities.

V. C. Pond has used all the well-known makes, and does not find that they differ much; finds decay to recur about margins of fillings, a large proportion wasting, and many discoloring the tooth-substances.

Chas. T. Allan says that, for filling in first molars of very poor and soft structure, it is the best material we have. Regards it almost as highly for proximal and buccal cavities in molar teeth of adults when of very poor structure, and when used in these cases does not think the washing away of material will prove an important factor.

G. L. Parmele has had good results in some cases; on the other hand, has been disappointed, first, by failure to harden and washing away; secondly, by extension of caries; does not employ it as much as formerly.

JOHN I. HART, D.D.S.,
*Editor New York Odontological Society.*

